# Silencing of the phytoene desaturase (*PDS*) gene affects the expression of fruit-ripening genes in tomatoes

**DOI:** 10.1186/s13007-019-0491-z

**Published:** 2019-10-04

**Authors:** Aung Htay Naing, Swum Yi Kyu, Phyo Phyo Win Pe, Kyeung Il Park, Je Min Lee, Ki Byung Lim, Chang Kil Kim

**Affiliations:** 1 Department of Horticultural Science, Kyungpook National University, Daegu, South Korea; 2 Department of Horticulture and Life Science, Yeungnam University, Gyeongsan, South Korea

**Keywords:** Carotenoid biosynthesis, Ethylene biosynthesis, Fruit ripening, Relative gene expression, VIGS

## Abstract

**Background:**

Past research has shown that virus-induced phytoene desaturase (*PDS*) gene silencing via agroinjection in the attached and detached fruit of tomato plants results in a pale-yellow fruit phenotype. Although the *PDS* gene is often used as a marker for gene silencing in tomatoes, little is known about the role of *PDS* in fruit ripening. In this study, we investigated whether the pepper *PDS* gene silenced endogenous *PDS* genes in the fruit of two tomato cultivars, Dotaerang Plus and Legend Summer.

**Results:**

We found that the pepper *PDS* gene successfully silenced endogenous *PDS* in tomato fruit at a silencing frequency of 100% for both cultivars. A pale-yellow silenced area was observed over virtually the entire surface of individual fruit due to the transcriptional reduction in phytoene desaturase (*PDS*), zeta-carotene (*ZDS*), prolycopene isomerase (*CrtlSO*), and beta-carotene hydroxylase (*CrtR*-*b2*), which are the carotenoid biosynthesis genes responsible for the red coloration in tomatoes. *PDS* silencing also affected the expression levels of the fruit-ripening genes Tomato AGAMOUS-LIKE1 (*TAGL1*), RIPENING INHIBITOR (*RIN*), pectin esterase gene (*PE*), lipoxygenase (*LOX*), FRUITFULL1/FRUITFUL2 (*FUL1/FUL2*), and the ethylene biosynthesis and response genes 1-aminocyclopropane-1-carboxylate oxidase 1 and 3 (*ACO1* and *ACO3*) and ethylene-responsive genes (*E4* and *E8*).

**Conclusion:**

These results suggest that *PDS* is a positive regulator of ripening in tomato fruit, which must be considered when using it as a marker for virus-induced gene silencing (VIGS) experiments in order to avoid fruit-ripening side effects.

## Background

Virus-induced gene silencing (VIGS) is a widely used reverse genetics tool for the high-throughput analysis of the biological functions of target genes in plants due to its ability to rapidly degrade the mRNA of the target gene, its simplicity in use, and its rapid results. Application of VIGS was first achieved with the silencing of the phytoene desaturase (*PDS*) gene in tobacco leaves, resulting in visible leaf photo-bleaching [1]. Since then, VIGS has been used to investigate the function of genes involved in a number of research areas, including abiotic and biotic defense mechanisms, coloration (i.e., anthocyanin and carotenoid biosynthesis), and plant growth [2–4], and more than 30 plant species have been subject to this form of investigation [5].

With the aim of optimizing the VIGS approach, *PDS* is widely used as a common marker gene in many plant species due to its ease of detection. The use of VIGS in tomatoes was first achieved by silencing *PDS* in leaves [6], followed by many more successful *PDS*-silencing experiments on tomato fruit [7–9]. The increase in carotenoid content (in the order of a 10- to 14-fold increase) during the ripening of tomatoes has been reported [10]. The *PDS* gene encoding the phytoene desaturase enzyme is involved in carotenoid biosynthesis pathway [10] and its silencing in the fruit of tomatoes reduced carotenoid accumulation and led to yellow-colored fruit [8, 9]. In addition, transcriptional control of *PDS* in carotenoid biosynthesis in tomato flowers and fruits has also been reported [11–13]. However, the molecular mechanism how the *PDS* silencing affects carotenoid biosynthesis in tomato fruits has not been well explored. Su et al. [14] reported that carotenoid accumulation during the ripening of tomatoes is controlled by the transcript levels of beta-carotene hydroxylase (*CrtR*-*b2*) gene. In addition, Fantini et al. [13] also claimed that reduction of carotenoid contents in tomato fruits is associated with down-regulation of the carotenoid biosynthesis genes *zeta*-carotene desaturase (*ZDS*) and prolycopene isomerase (*CrtISO*). Hence, we investigated whether *PDS* silencing influences the regulation of the above mentioned genes involved in carotenoid biosynthesis pathway.

Previously, Kim et al. [15] reported that the silencing of the pepper *PDS* gene, which has high homology to the *PDS* gene in tomato and petunia plants, in pepper leaves and fruit led to distinct photo-bleaching. Recently, Naing et al. [16] reported that the pepper *PDS* gene significantly suppressed endogenous *PDS* gene in petunia and reduced total chlorophyll content. Hence, pepper *PDS* was exploited to silence the endogenous *PDS* gene in tomato fruit. Based on our preliminary work, the silencing of the endogenous *PDS* gene in detached tomatoes led to fruit with a yellow color that continued until the fruit had softened. Vrebalov et al. [17] observed that the reduction of the RIPENING INHIBITOR (*RIN*) gene results in the failure of fruit to ripen. In addition, Vrebalov et al. [18] *and* Itkin et al. [19] also claimed that Tomato AGAMOUS-LIKE1 (*TAGL1*) is involved in the regulation of fruit ripening and its suppression results in yellow-orange fruits, decreased carotenoids and delayed ripening. Moreover, the involvement of pectin esterase gene (PE), lipoxygenase (LOX), and the two homologous genes FRUITFULL1 (*FUL1*) and FRUITFUL2 (*FUL2*) in the ripening of tomatoes has been identified [17, 19–21]. In the present study, we were interested in investigating whether the expression patterns of the fruit-ripening related genes would change in *PDS*-silenced tomato fruits.

Because tomatoes are climacteric fruit, their ripening is associated with the expression of ethylene biosynthesis genes such as 1-aminocyclopropane-1-carboxylate oxidase (*ACO*) [20, 22–24]. Alexander and Grierson [25] and Hu et al. [26] reported that the suppression of *SlACO1* delayed fruit ripening and ethylene biosynthesis, while the transcript levels of *SlACO1* and *SlACO3* are markedly high when the ripening of tomatoes is triggered [25, 27]. Similarly, Lincoln et al. [28] and Zhang et al. [20] observed that expression of *E4* is positively associated with ethylene biosynthesis, while Kneissl and Deikman [29] also reported that *E8* is a fruit ripening-specific gene that is activated during the fruit ripening process, and it is widely used as a fruit-specific promoter in transgenic tomatoes [30, 31].

In summary, in the present study, *PDS* expression was silenced in the fruit of two commercial tomato cultivars using VIGS to elucidate how *PDS* silencing affects the fruit phenotype and the expression of the genes mentioned above that are involved in the ripening of tomatoes.

## Materials and methods

### Plant materials

Seeds of two tomato cultivars, Dotaerang Plus (Takii Korea Co., Ltd.) and Legend Summer (Haesung Seed Plus Co., Ltd.), were sown in plug trays filled with the soil-less mixture BM7 (Berger Co., Quebec, Canada) in a growth chamber at 23 °C, with a 16 h photoperiod (400 µmol m^−2^ s^−1^) and 70% relative humidity (RH). After 2 weeks, healthy germinated plants of uniform size were transplanted into pots filled with BM7 and moved to a greenhouse at 25–27 °C (daytime) and 16–18 °C (night), with a 16-h photoperiod and 70% RH for plant growth and fruiting. When the fruit had reached the mature green stage, healthy fruits of uniform size were picked at 8 AM from each cultivar and used for VIGS testing.

### Preparation of *Agrobacterium* suspension

The use of the tobacco rattle virus (TRV1 and TRV2) in VIGS experiments has been reported for tomatoes [6]. *Agrobacterium tumefaciens* strain GV3101 harboring the tobacco rattle virus vectors pTRV1, pTRV2 (without *PDS*), and pTRV2-*PDS* was provided by Prof. Je Min Lee (Kyungpook National University, Daegu), while the *PDS* gene inserted into the vector pTRV2-*PDS* was cloned from pepper. Bacterial samples each harboring one of the three vectors were separately cultured in LB broth containing 25 mg L^−1^ kanamycin and rifampicin in a shaking incubator at 300 rpm and 28 °C. When the optical density of the bacteria was OD_600_ 0.6, the cells were centrifuged and the obtained pellets suspended in an inoculation buffer of 10 mM MgCl_2_, 10 mM MES (pH 5.6), and 200 µM acetosyringone, to obtain a final OD_600_ of 1.0 for each culture. The cells were then placed in a rotary shaker at 300 rpm and 28 °C for 6 h.

### Agroinjection into detached tomatoes

**Fig. 1:**
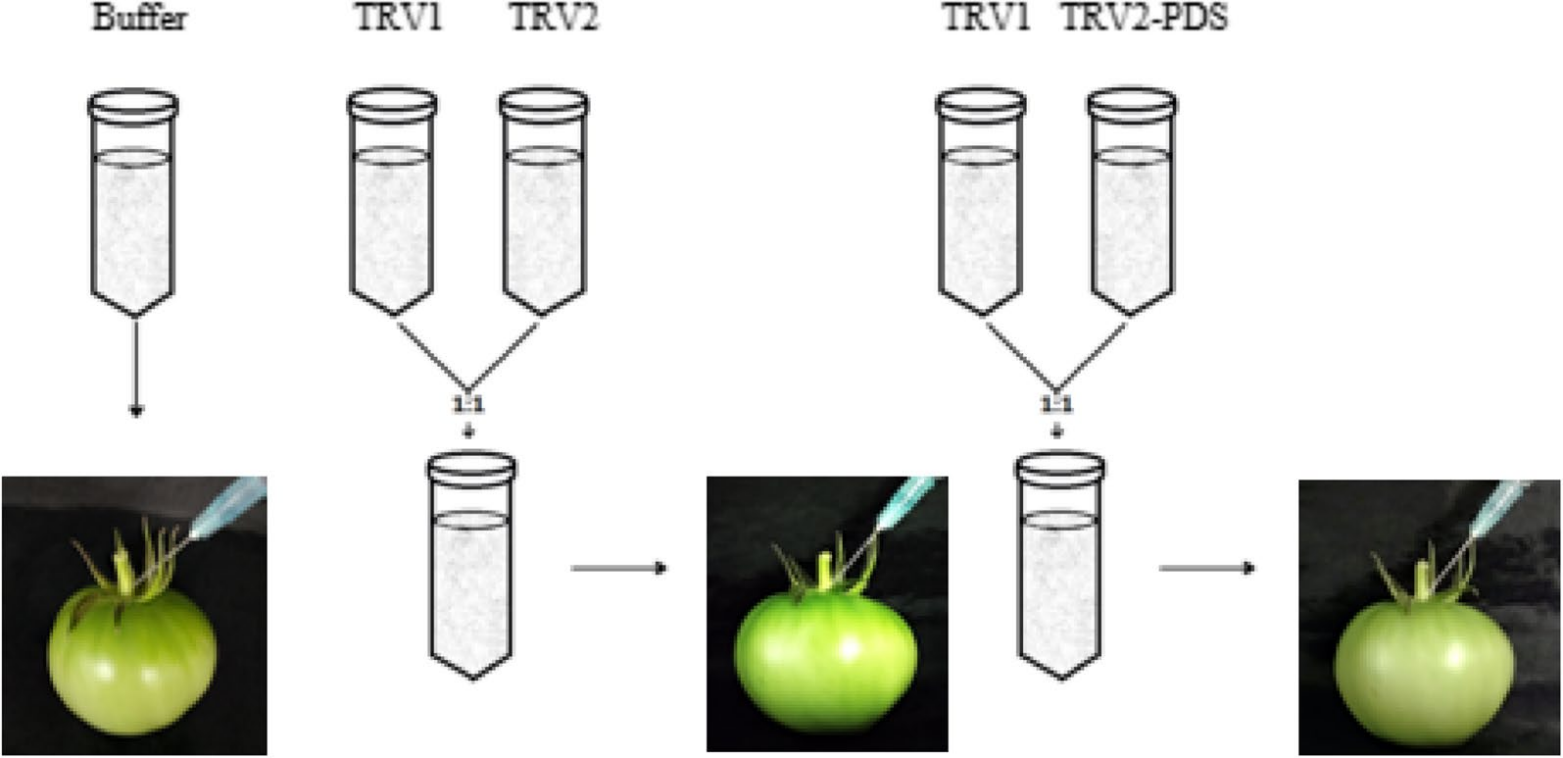
Schematic of injections to the carpopodium of green mature tomato fruits using a 1 mL syringe

Green mature tomatoes were harvested at 8 AM from each cultivar and immediately divided into three groups to be separately injected with a buffer solution alone or one of the bacterial suspensions, pTRV1 + pTRV2 (TRV1/2) or pTRV1 + pTRV2-*PDS* (TRV1/2-*PDS*; Fig. 1). The suspensions were injected through the carpopodium with a 1-mL syringe at room temperature. Once the suspension had infiltrated the tissue, it appeared in the sepals. There were 20 tomatoes per treatment and three replications of each treatment. The injected fruits were placed in a growth chamber at 23 °C, with a 16-h photoperiod (400 µmol m^−2^ s^−1^) and 70% relative humidity.

### Evaluation of fruit phenotype

Two weeks after injection, the presence of pale yellow and red coloring of the pericarp was evaluated for both cultivars. To determine the silencing frequency, the following calculation was used:

$$ {\text{Silencing}}\;{\text{frequency }}\left( \% \right) \, = {\text{ number}}\;{\text{of}}\;{\text{tomatoes}}\;{\text{with}}\;{\text{visible}}\;{\text{silencing}}/{\text{total}}\;{\text{number}}\;{\text{of}}\;{\text{tomatoes}}\;{\text{injected}} \times 100 $$

### Measurement of gene expression levels using quantitative real time-PCR

Two weeks after injection, RNA was extracted from the pericarp of the tomatoes injected with the different inoculums; for those injected with TRV1/2-*PDS*, RNA was extracted from visibly silenced areas. Total RNA extraction was performed using an RNAqueous kit (Ambion Inc., Austin, TX, USA). Reverse transcription was conducted using 1 µg of total RNA and an oligo dT20 primer with a ReverTra Ace-α kit (Toyobo Co. Ltd., Osaka, Japan). Transcript levels of the endogenous tomato *PDS* gene, the carotenoid biosynthesis genes (*ZDS*, *CrtR*-*b2*, and *CrtlSO*), the fruit-ripening-associated genes (*FUL1, FUL2, PE, LOX, TAGL1,* and *RIN*), and the ethylene biosynthesis and response genes (*ACO1*, *ACO3, E4,* and *E8*) were measured using a StepOnePlus Real-Time PCR system (Thermo Fisher Scientific Inc., Waltham, MA, USA). Expression levels were normalized to the actin gene to minimize variation in the cDNA template. The primers, accession numbers, and PCR conditions for the examined genes are listed in Additional file 1: Table S1.

### Statistical analysis

For qRT-PCR analysis, total RNA was extracted from three different biological samples, and this analysis was repeated three times for each biological sample. The data were analyzed using SPSS v.11.09 (IBM Corporation, Armonk, NY, USA), and the values of the relative gene expression levels described in the study were means of the three biological replicates. The least significant difference test (LSDT) was used to identify differences between the means. A *P*-value of less than 0.05 was considered statistically significant.

## Results

### *PDS*-silencing fruit phenotype

**Fig. 2:**
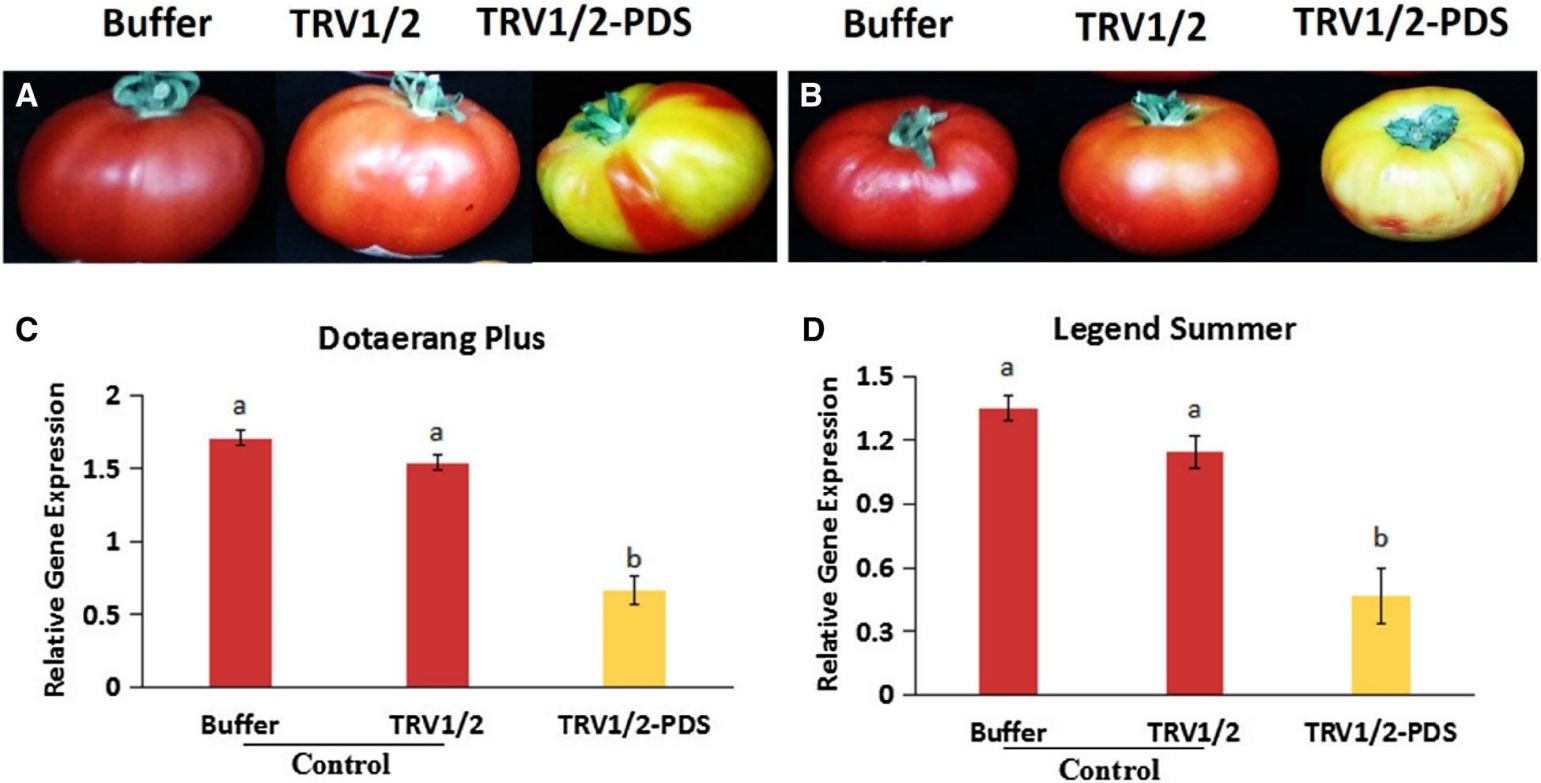
Silencing of *PDS* in green mature fruits of two different tomato cultivars (Dotaerang Plus and Legend Summer). Different fruit phenotypes of cvs. Dotaerang Plus (**A**) and Legend Summer (**B**) injected with buffer, TRV1/2, and TRV1/2-*PDS*. Differences in transcript levels of the *PDS* gene in cvs. Dotaerang Plus (**C**) and Legend Summer (**D**) injected with buffer, TRV1/2, and TRV1/2-*PDS*. Data represent the mean of three biological replicates, and the bar indicates the standard deviation. Means with different letters are statistically significant (*LSDT*, *P < 0.05)

Mature green tomatoes harvested from the two cultivars (Dotaerang Plus and Legend Summer) were immediately injected with the buffer, TRV1/2, or TRV1/2-*PDS*. Two weeks after injection, silenced phenotypes associated with *PDS* were observed in the TRV1/2-*PDS* tomatoes, but not in those injected with the buffer or TRV1/2. A distinct pale-yellow coloration was observed in fruits of both cultivars injected with TRV1/2-*PDS* (Fig. 2A, B). In this study, all tomatoes injected with TRV1/2-*PDS* exhibited a silencing efficiency of 100%, and the silencing patterns of the fruit within the same cultivar were also similar based on visual observations (data not shown). The silenced areas of cv. Legend Summer covered virtually the entire fruit, while this was not observed in cv. Dotaerang Plus (Fig. 2A, B), indicating that there is variation in the effectiveness of silencing depending on the cultivars used. Tomatoes injected with the buffer solution or TRV1/2, which were used as controls, did not exhibit any silencing symptoms and developed the customary red color, although those tomatoes injected with the buffer solution were a deeper red compared to those injected with TRV1/2 for both cultivars (Fig. 2A, B).

### Expression of the *PDS* gene

To clarify whether the silencing of *PDS* was associated with the phenotypic variation, the transcript levels of *PDS* expressed in the pericarp of the injected fruit were determined using quantitative real time-PCR (qRT-PCR). For both cultivars, the transcript levels detected in tomatoes injected with TRV1/2-*PDS* (pale yellow) were significantly lower than those of the control groups (red), while the expression levels in tomatoes injected with TRV1/2 were slightly lower than those injected with the buffer (Fig. 2C, D). These results indicate that VIGS significantly inhibits *PDS* expression, which is consequently associated with the observed fruit phenotypes.

### Expression of carotenoid biosynthesis genes

**Fig. 3:**
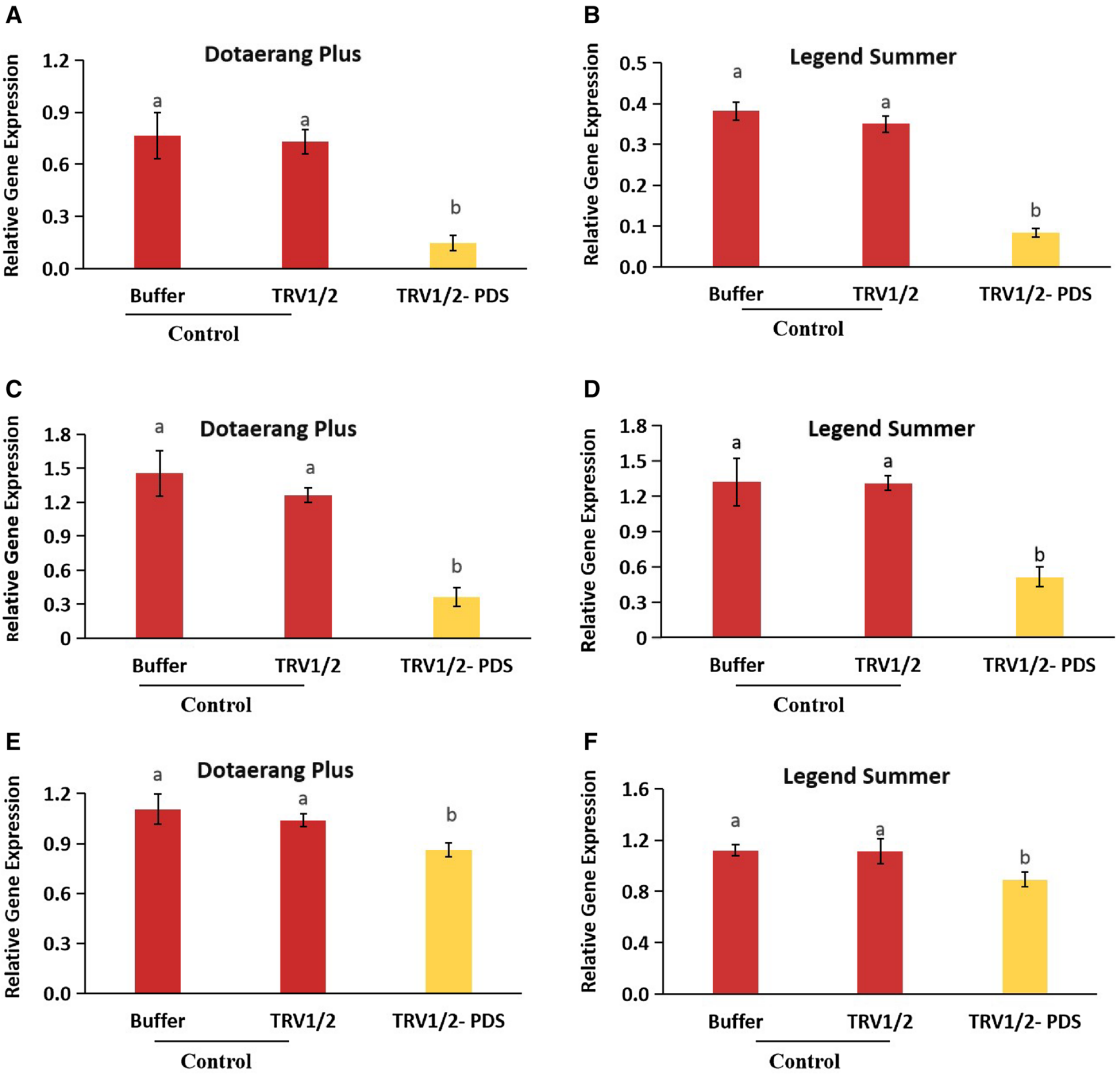
Differences in transcript levels of the carotenoid biosynthesis genes, *ZDS* (**A**, **B**), *CrtR*-*b2* (**C**, **D**), and *CrtlSO* (**E**, **F**) expressed in cvs. Dotaerang Plus and Legend Summer injected with buffer, TRV1/2, and TRV1/2-*PDS*. Data represent the means of three biological replicates, and the bar indicates the standard deviation. Means with different letters are statistically significant (*LSDT*, *P < 0.05)

Because *PDS* encodes a key enzyme involved in the carotenoid biosynthesis pathway, its silencing may affect carotenoid accumulation. We investigated whether *PDS* silencing affected carotenoid accumulation in tomatoes by measuring the expression levels of the carotenoid biosynthesis genes (*ZDS, CrtR*-*b2, and CrtlSO*), which are involved in carotenoid biosynthesis, using qRT-PCR (Fig. 3A–F). Silencing of the *PDS* gene significantly reduced the expression levels of the biosynthesis genes compared to the control groups for both cultivars, which could lead to a reduction in the carotenoids in *PDS*-silenced tomatoes. This association is supported by the visual appearance of the experimental tomatoes, which appeared to have lower levels of carotenoids than the control fruit.

### Expression of fruit-ripening genes

**Fig. 4:**
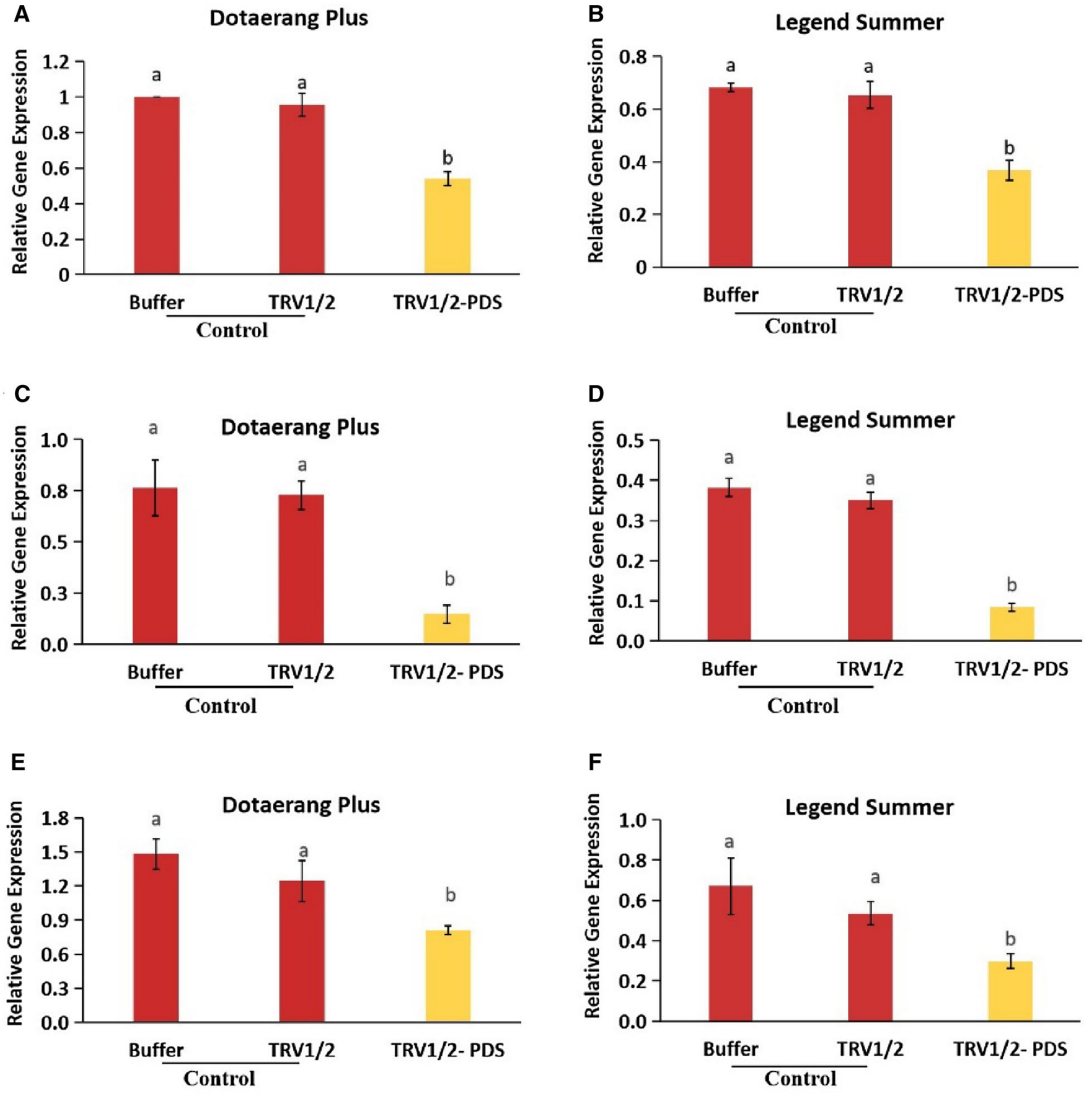

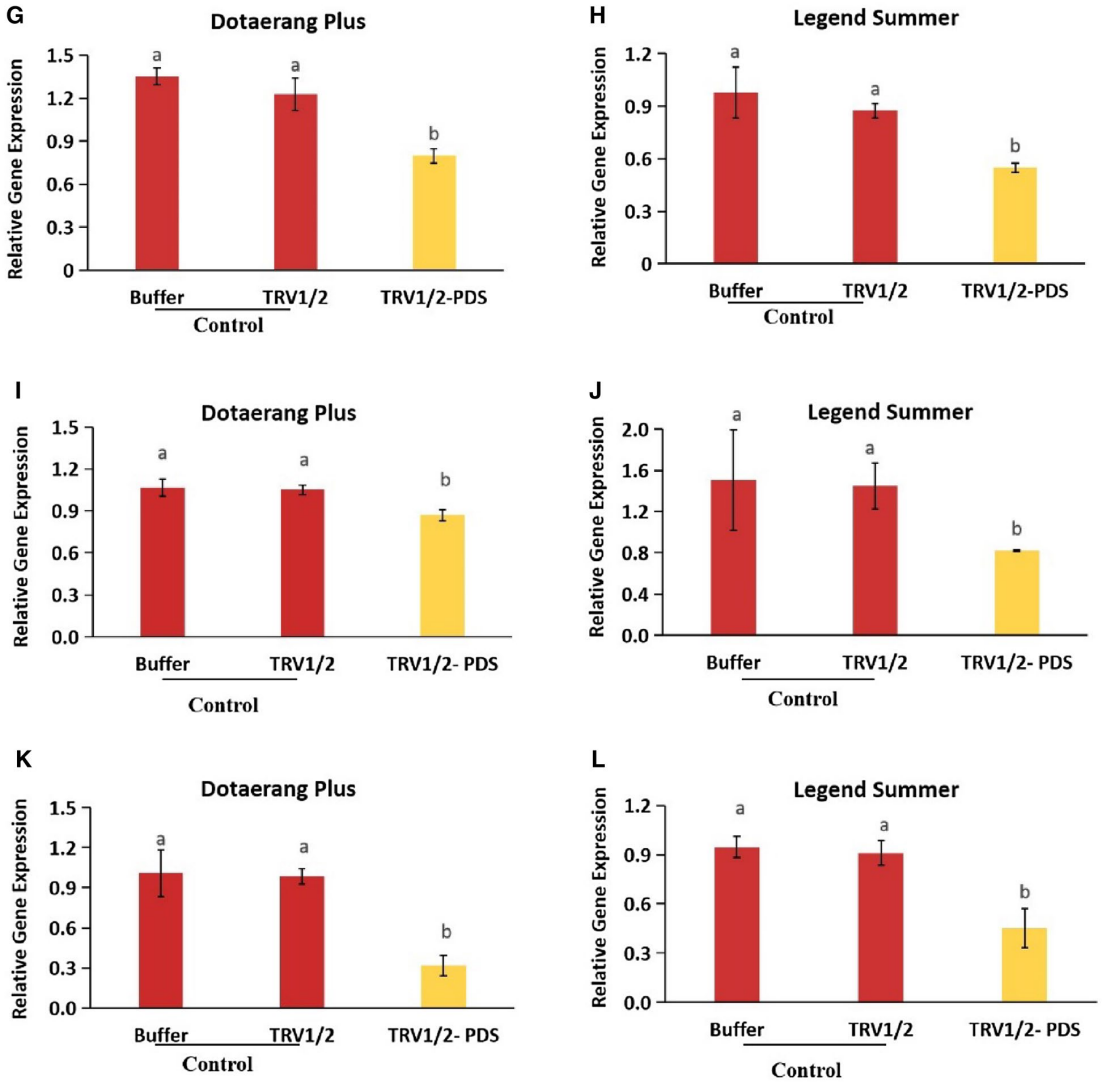
Differences in transcript levels of the fruit ripening genes, *FUL1* (**A**, **B**), *FUL2* (**C**, **D**), *LOX* (**E**, **F**), *RIN* (**G**, **H**), *PE* (**I**, **J**), and *TAGL1* (**K**, **L**) expressed in cvs. Dotaerang Plus and Legend Summer injected with buffer, TRV1/2, and TRV1/2-*PDS.* Data represent the means of three biological replicates, and the bar indicates the standard deviation. Means with different letters are statistically significant (*LSDT*, *P < 0.05)

In this study, *PDS*-silenced tomatoes did not ripen, remaining pale yellow until the fruit had softened, while the control tomatoes underwent natural ripening, with softening occurring approximately 10 days earlier than in the *PDS*-silenced tomatoes. The expression levels of the known ripening genes (*TAGL1*, *RIN, PE, LOX, FUL1,* and *FUL2*) were analyzed for all tomatoes and found to be significantly down-regulated in the *PDS*-silenced fruits compared to the controls (Fig. 4A–L). These results suggest that *PDS* might influence the ripening of tomatoes by regulating the expression of these genes.

### Expression of ethylene biosynthesis and response genes

**Fig. 5:**
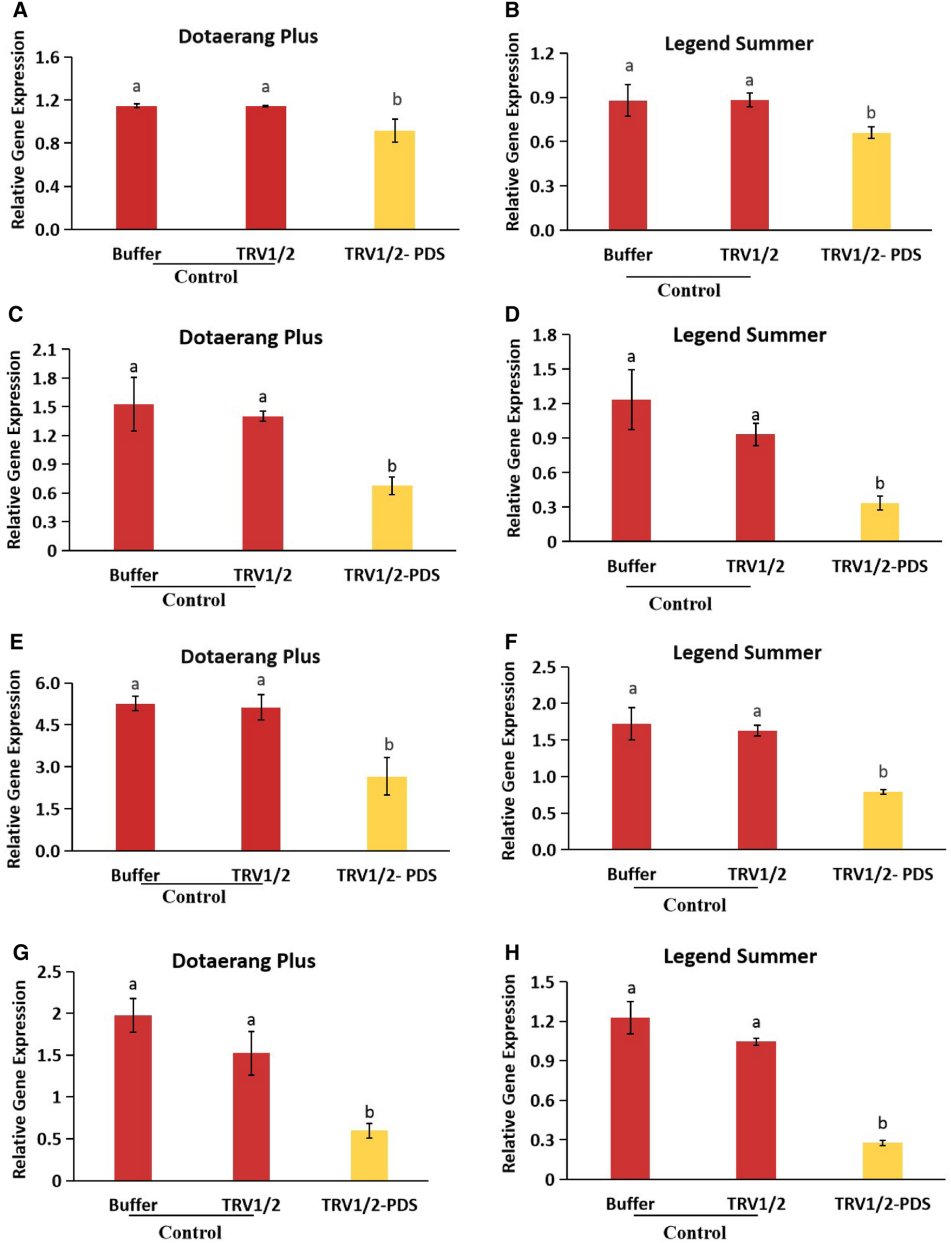
Differences in transcript levels of the ethylene biosynthesis and response genes, *ACO1* (**A**, **B**), *ACO3* (**C**, **D**), *E4* (**E**, **F**), and *E8* (**G**, **H**) expressed in cvs. Dotaerang Plus and Legend Summer injected with buffer, TRV1/2, and TRV1/2-*PDS*. Data represent the means of three biological replicates, and the bar indicates the standard deviation. Means with different letters are statistically significant (*LSDT*, *P < 0.05)

Tomatoes are climacteric, meaning their ripening is associated with the transcriptional activation of ethylene biosynthesis and response genes. We further explored the impact of reduced *PDS* mRNA levels on ethylene biosynthesis during the ripening process by determining the expression levels of the ethylene biosynthesis genes (*ACO1* and *ACO3*) and response genes (*E4* and *E8*). Results indicated that these genes were significantly down-regulated in *PDS*-silenced fruits compared to the controls (Fig. 5A–H). These results suggest that the silencing of *PDS* might inhibit the transcription of these genes.

## Discussion

The silencing of *PDS* using VIGS has been reported for a variety of plants, including tomato, pepper, and eggplant leaves, that belong to the Solanaceae family [6, 32, 33]. In these studies, *PDS* was used as a marker gene due to the photo-bleaching caused by its inactivation. However, only a few reports have described the silencing of *PDS* in the fruit of tomatoes [8, 9], and they did not examine *PDS* gene silencing beyond photo-bleaching. Because *PDS* encodes an enzyme involved in the carotenoid biosynthesis pathway, it is interesting to investigate whether its silencing would inhibit the expression of carotenoid biosynthesis genes and those involved in fruit ripening and ethylene biosynthesis.

In this study, all tomato fruit of both cultivars (Legend Summer and Dotaerang Plus) injected with TRV1/2-*PDS* exhibited a pale yellow phenotype, representing a significant difference in appearance compared to non-*PDS*-silenced fruits (Fig. 2A, B). Expectedly, the exploitation of the *PDS* gene to silence the endogenous *PDS* gene in the tomato cultivars resulted in similar phenotypes to those reported by Orzaez et al. [8], Wang et al. [34], and Romero et al. [9], who silenced *PDS* in attached and detached tomatoes (cvs. Micro Tom, Maxifort, Jinfen) using the tomato *PDS* gene. In the control fruits, those injected with a buffer exhibited a deeper red color than those injected with TRV1/2. It is likely that “TRVI/2” itself is able to degrade *PDS* mRNA to some extent because the transcript levels of *PDS* were slightly lower in the TRVI/2-treated tomatoes than in the buffer-treated fruit. The slight differences observed in the *PDS*-silenced areas of the two different cultivars could be due to genetic differences. A significant reduction in *PDS* transcript levels was observed in *PDS*-silenced tomatoes compared to those of the controls, as illustrated by the resultant fruit phenotypes. Orzaez et al. [8] and Romero et al. [9] reported similar findings in their studies. These results suggest that the pepper *PDS* gene was able to silence the endogenous *PDS* gene in the tomato cultivars.

Su et al. [14] observed that carotenoid accumulation during the ripening of tomatoes is linked to the transcript levels of *CrtR*-*b2* genes. In addition, the association between the carotenoid accumulation and upregulation of *PSY1, ZDS, CrtlSO* had also been reported in tomato [11–13]. In this study, the transcript levels of the carotenoid biosynthesis genes *ZDS, CrtR*-*b2, and CrtlSO* were also significantly lower in the *PDS*-silenced fruits than in the controls; these lower transcript levels were likely to prevent the accumulation of enough carotenoids to produce a red color in the fruit, as indicated by the lack of red in the *PDS*-silenced tomatoes. The lower accumulation of carotenoids resulting from the silencing of *PDS* in the fruit of tomatoes has been reported previously, although the transcription levels of these biosynthesis genes were not specifically examined [8, 9, 13].

The downregulation of the fruit-ripening genes *TAGL1*, *RIN, PE, LOX, FUL1,* and *FUL2* was also observed in the *PDS*-silenced tomatoes but not in the controls, suggesting that *PDS* silencing inhibited fruit ripening. Downregulation of these genes resulting in defective ripening with fruit softening has also been reported in previous research [18, 19, 35–37]. In addition, Bemer et al. [36] and Shima et al. [37] also reported that the tomato *FUL1 and FUL2* genes participate in fruit ripening via their interaction with *RIN*. *FUL1/FUL2* and *RIN* genes code for enzymes upstream of the ethylene signaling pathway during ripening, and suppression of these genes results in a ripening-defective phenotype with lower ethylene production [18, 37–39]. Because the downregulation of the *FUL1/FUL2* and *RIN* genes was observed in *PDS*-silenced tomatoes in this study, we also expected that the downregulation of the ethylene biosynthesis and response genes *ACO1*, *ACO3*, *E4,* and *E8* would occur, and our analysis revealed that these genes were indeed downregulated in *PDS*-silenced fruit. Zhang et al. [20] also suggested that the transcription levels of *PDS* in tomato fruit were positively associated with those of other genes, such as *TAGL1*, *FUL1/FUL2, RIN, ACO1, ACO3, E4,* and *E8,* that are involved in fruit ripening. Taken together, we conclude that the silencing of the *PDS* gene led to the downregulation of ethylene biosynthesis and response genes and fruit-ripening genes, leading to a ripening-defective phenotype. In some VIGS studies of fruit, *PDS* was used as marker gene to characterize the functional role of target genes; for example, Orzaez et al. [40] identified the specific role of *Delia* (D) and *Rosea* (R) in anthocyanin accumulation in tomatoes with the combined construct TRV2:DR:PDS. However, it is relatively difficult to specifically characterize the function of a target gene due to potential side effects of *PDS* in terms of fruit pigmentation. Our results suggest that these effects should be taken into account when using *PDS* as a marker gene in the VIGS-based research of fruit.

## Conclusion

We demonstrated that the pepper *PDS* gene effectively silenced transcription of the endogenous *PDS* gene in the fruit of two tomato cultivars, and this silencing affected the regulation of the *ZDS, CrtlSO,* and *CrtR*-*b2* genes, which are involved in the carotenoid biosynthesis pathway. *PDS*-silencing also appeared to affect fruit ripening, acting as a positive regulator by modulating the fruit-ripening genes *TAGL1*, *RIN, PE, LOX, FUL1,* and *FUL2* and their associated ethylene biosynthesis and response genes *ACO1*, *ACO3, E4,* and *E8*. These results suggest that *PDS* silencing not only affects the carotenoid pathway but also leads to the inhibition of other genes involved in the fruit-ripening process in tomatoes. We expect that our work will aid in the better understanding of the regulatory mechanisms of *PDS* in the fruit-ripening process.

## Supplementary information

**Additional file 1: Table S1.** Primers, accession numbers, and PCR conditions used for qRT-PCR analysis of the genes.

## Data Availability

The datasets used and/or analyzed during the current study are available from the corresponding authors on reasonable request.

## Abbreviations

*ACO1*: 1-aminocyclopropane-1-carboxylate oxidase 1
*ACO3*: 1-aminocyclopropane-1-carboxylate oxidase 3
*CrtR*-*b2*: beta-carotene hydroxylase
*CrtlSO*: prolycopene isomerase
*E4, E8*: ethylene responsive gene
*FUL1*: FRUITFULL1
*FUL2*: FRUITFULL2
LOX: lipoxygenase
*PDS*: phytoene desaturase
*PE*: pectin esterase gene
*RIN*: RIPENING INHIBITOR
*TAGL1*: Tomato AGAMOUS-LIKE1
TRV1: tobacco rattle virus 1
TRV2: tobacco rattle virus 2
VIGS: virus-induced gene silencing
ZDS: zeta-carotene
