# Supplementary material for: Silencing of the phytoene desaturase (PDS) gene affects the expression of fruit-ripening genes in tomatoes
Source: Plant Methods. 2019 Oct 4;15:110. doi: 10.1186/s13007-019-0491-z (PMC6777038; doi:10.1186/s13007-019-0491-z)
Supplement: Supplementary file 1 — Additional file 1: Table S1. Primers, accession numbers, and PCR conditions used for qRT-PCR analysis of the genes. [file 13007_2019_491_MOESM1_ESM.docx]

Table S1 Primers, accession numbers, and PCR conditions used for qRT-PCR analysis of the genes

| **Gene** | **Gene Accession Number** | **Primer sequence (5' to 3')** | **PCR condition** |
| --- | --- | --- | --- |
| ***PDS*** | NM_001247166.2 | **F:** GCCAAATCACCGAAAGCAGG  **R:** TGGTTTGGCAGTTACCCAGA | 95˚C (10 min) →[95˚C (15s) →58˚C (30s) ] x 40cycles→95˚C (15s) →60 ˚C(1min) →95 ˚C(15sec) |
| ***CRTISO*** | NM_001309230.1 | **F:** GGAATAGGTGGATTGGTGGCA  **R**: ACCATCCCTCTCGTAAAAGCC | 95˚C (10 min) →[95˚C (15s) →60˚C (30s) ] x 40cycles→95˚C (15s) →60 ˚C(1min) →95 ˚C(15sec) |
| ***CrtR-b2*** | NM_001279052.2 | **F:** AGAAAAGGAAGTCAACCGAAGGA  **R:** ATCTACAGCCTAAATACAATCGC | 95˚C (10 min) →[95˚C (15s) →58˚C (30s) ] x 40cycles→95˚C (15s) →60 ˚C(1min) →95 ˚C(15sec) |
| ***ZDS*** | NM_001247454.2 | **F:** GGGTCACCTGGATTCTTGGTT  **R:** ATCAGCCCGAATAACCGACTT | 5˚C (10 min) →[95˚C (15s) →59˚C (30s) ] x 40cycles→95˚C (15s) →60 ˚C(1min) →95 ˚C(15sec) |
| ***ACO1*** | NM_001247095.2 | **F:** GTAAGGGACTTGAGGCTGTTCA  **R:** AGTAGGAAGATGGCGCAAGAA | 95˚C (10 min) →[95˚C (15s) →59˚C (30s) ] x 40cycles→95˚C (15s) →60 ˚C(1min) →95 ˚C(15sec) |
| ***ACO3*** | NM_001309213.1 | **F:** CGCCAAAAGACAGGGCTAAA  **R:** AAAACCCCATTTCTCCGATGC | 95˚C (10 min) →[95˚C (15s) →58˚C (30s) ] x 40cycles→95˚C (15s) →60 ˚C(1min) →95 ˚C(15sec) |
| ***E4*** | S44898.1 | **F:** TGATGTGGGAAAGCAATACCG  **R:** CTTCTAACGACTCCCTTGCCA | 95˚C (10 min) →[95˚C (15s) →59˚C (30s) ] x 40cycles→95˚C (15s) →60 ˚C(1min) →95 ˚C(15sec) |
| ***E8*** | X13437.1 | **F:**  CCGCCAAAAGACAGGGCTAA  **R:** AAACCCCATTTCTCCGATGC | 95˚C (10 min) →[95˚C (15s) →58˚C (30s) ] x 40cycles→95˚C (15s) →60 ˚C(1min) →95 ˚C(15sec) |
| ***RIN*** | NM_001247741.2 | **F:** ATGTACAACCCGAAAATGCAGC  **R:** ACTCCAAATTCAAAGCATCCATCC | 95˚C (10 min) →[95˚C (15s) →59˚C (30s) ] x 40cycles→95˚C (15s) →60 ˚C(1min) →95 ˚C(15sec) |
| ***LOX*** | NM_001246883.2 | **F: AGGTGTTAAAGCTGTGGTGACT**  **R: AGCTCAGCAGCAACAATCCAT** | 95˚C (10 min) →[95˚C (15s) →58˚C (30s) ] x 40cycles→95˚C (15s) →60 ˚C(1min) →95 ˚C(15sec) |
| ***FUL1*** | NM_001247244.2 | **F:** CTTCCGTCTGTCTCCATTTGAT  **R:** TCGCTTCAACTGGACTCTTCC | 95˚C (10 min) →[95˚C (15s) →59˚C (30s) ] x 40cycles→95˚C (15s) →60 ˚C(1min) →95 ˚C(15sec) |
| ***FUL2*** | NM_001307938.1 | **F:** GCATACCCAAATGCAGGAGAC  **R:** TGAGATGGCGAAGCATCCAT | 95˚C (10 min) →[95˚C (15s) →59˚C (30s) ] x 40cycles→95˚C (15s) →60 ˚C(1min) →95 ˚C(15sec) |
| ***PE*** | X74638.1 | **F:** GCTGAGTGGGACGGAGATTT  **R:** TAACCAGGCCACTTGACACG | 95˚C (10 min) →[95˚C (15s) →59˚C (30s) ] x 40cycles→95˚C (15s) →60 ˚C(1min) →95 ˚C(15sec) |
| ***TAGL1*** | NM_001313930.1 | **F:** GCAAGCGTAGAAATGGGCTAT  **R:** ATTGGCATATTCATAGAGACGGC | 95˚C (10 min) →[95˚C (15s) →58˚C (30s) ] x 40cycles→95˚C (15s) →60 ˚C(1min) →95 ˚C(15sec) |
| ***Actin*** | NM_001330119.1 | **F:** GGGACGTGAAAGAAAAGCTCG  **R:** GGAAACGCTCAGCACCAATG | 95˚C (10 min) →[95˚C (15s) →59.5˚C (30s) ] x 40cycles→95˚C (15s) →60 ˚C(1min) →95 ˚C(15sec) |
